# Letter to the editor regarding “elevated C-reactive protein to albumin ratio is a promising predictive biomarker for prognosis in patients with renal cell carcinoma”

**DOI:** 10.1080/07853890.2026.2637270

**Published:** 2026-03-07

**Authors:** Gizem Zorlu Gorgulugil, Muhammed Ali Coskuner, Gokhan Koker

**Affiliations:** a Department of Internal Medicine, University of Health Sciences, Antalya Training and Research Hospital, Antalya, Turkey; b Department of Internal Medicine, University of Health Sciences, Antalya City Hospital, Antalya, Turkey

Dear Editor

We read with great interest the recent meta-analysis by Xu et al. entitled ‘*Elevated C-reactive protein to albumin ratio is a promising predictive biomarker for prognosis in patients with renal cell carcinoma*’, published in *Annals of Medicine* [1]. By synthesizing data from 10 studies involving 2,478 patients, the authors provide a timely and clinically relevant overview of the prognostic significance of the C-reactive protein to albumin ratio (CAR) in renal cell carcinoma (RCC).

One of the major strengths of this study is the comprehensive evaluation of prognostic performance using multiple diagnostic metrics, including sensitivity, specificity, diagnostic odds ratio, and summary receiver operating characteristic (SROC) analysis. The reported pooled AUC of 0.77 suggests that CAR offers a meaningful, albeit moderate, discriminative ability. Importantly, the use of CAR as a continuous variable represents a methodological advantage over categorical inflammation-based scores such as the Glasgow Prognostic Score (GPS) or modified GPS, as it preserves biological variability and minimizes information loss [2].

Nevertheless, several limitations warrant consideration. First, most of the included studies were retrospective in nature, which inherently introduces selection bias and limits causal inference. Second, there was substantial heterogeneity in the reported CAR cut-off values (ranging from 0.025 to 0.11), highlighting the absence of standardized thresholds and complicating clinical implementation. Third, the majority of the included cohorts originated from East Asia, raising concerns regarding the generalizability of the findings to Western populations and other ethnic groups.

Moreover, although CAR appears to be a robust prognostic biomarker, its role as a *predictive* marker in the context of contemporary RCC therapies remains unclear. In the era of immune checkpoint inhibitors, tyrosine kinase inhibitors, and HIF-2α inhibitors, distinguishing prognostic from predictive utility is of critical importance [3]. Currently, it remains uncertain whether CAR can inform treatment selection or reflect therapeutic responsiveness, rather than merely indicating baseline disease aggressiveness.

Future investigations should therefore focus on prospective, multicenter validation studies with standardized CAR cut-off values. Integrating CAR into multivariable prognostic models alongside TNM staging, IMDC risk classification, and molecular biomarkers may further enhance risk stratification [4]. In addition, assessing *dynamic changes* in CAR during treatment could provide valuable insights into treatment response and survival outcomes. Finally, translational studies elucidating the biological mechanisms underlying the inflammation–nutrition axis reflected by CAR would strengthen its biological plausibility and clinical relevance [5].

In conclusion, the meta-analysis by Xu et al. underscores the potential of CAR as a promising prognostic biomarker in RCC. However, standardized methodologies, prospective validation, and treatment-specific analyses are required before CAR can be routinely incorporated into clinical decision-making.

## Data Availability

Data sharing is not applicable to this article as no new data were created or analysed in this research.

## References

[1] Xu C, Guo Y, Chen X, et al. Elevated C-reactive protein to albumin ratio is a promising predictive biomarker for prognosis in patients with renal cell carcinoma. Ann Med. 2026;58(1):2618431. doi:10.1080/07853890.2026.2618431. 41566198 PMC12829417

[2] Makino T, Izumi K, Iwamoto H, et al. Comparison of the prognostic value of ınflammatory and nutritional ındices in nonmetastatic renal cell carcinoma. Biomedicines. 2023;11(2):533. doi:10.3390/biomedicines11020533. 36831069 PMC9953714

[3] Choueiri TK, Powles T, Peltola K, et al. Belzutifan versus everolimus for advanced renal-cell carcinoma. N Engl J Med. 2024;391(8):710–721. doi:10.1056/NEJMoa2313906. 39167807

[4] Wu M, Zhou Y, Chen Q, et al. Prognostic role of pretreatment c-reactive protein to albumin ratio in urological cancers: a systematic review and meta-analysis. Front Oncol. 2022;12:879803. doi:10.3389/fonc.2022.879803. 35480099 PMC9035789

[5] Nakayama T, Takeshita H, Kagawa M, et al. Prognostic significance of inflammatory markers in patients with advanced renal cell carcinoma receiving nivolumab plus ipilimumab. Int J Clin Oncol. 2024;29(10):1528–1537. doi:10.1007/s10147-024-02593-1. 39046676

